# Performance of a Whole-Blood Interferon-Gamma Release Assay with *Mycobacterium* RD1-Specific Antigens among HIV-Infected Persons

**DOI:** 10.1155/2011/325295

**Published:** 2010-08-10

**Authors:** Akira Fujita, Atsushi Ajisawa, Nobuyuki Harada, Kazue Higuchi, Toru Mori

**Affiliations:** ^1^Department of Pulmonary Medicine, Tokyo Metropolitan Tama Medical Center, 2-8-29 Musashidai, Fuchu-shi, Tokyo 183-8524, Japan; ^2^Department of Infectious Diseases, Tokyo Metropolitan Cancer and Infectious Diseases Center Komagome Hospital, 18-22-3 Honkomagome, Bunkyo-ku, Tokyo 113-8677, Japan; ^3^The Research Institute of Tuberculosis, Japan Anti-Tuberculosis Association, 3-1-24 Matsuyama, Kiyose-shi, Tokyo 204-8533, Japan

## Abstract

*Objective*. To evaluate the usefulness of one of IGRAs, QuantiFERON-TB Gold (QFT-G), in human immunodeficiency virus- (HIV- ) infected patients with various CD4^+^ T cell counts. *Methods*. The QFT-G assay was performed using QFT-G kits among 107 HIV-infected patients including 9 cases with active tuberculosis (TB). *Results*. In HIV-infected patients with CD4^+^ > 50/*μ*L, QFT-G positive rate for active TB patients was 5/6 (sensitivity = 83%), and that for those without active disease was 1/69 (specificity = 99%). The frequency of indeterminate QFT-G test was significantly higher in those with CD4^+^ less than 50/*μ*L (*P* < .0001). At the same time there was a proportional relationship between CD4^+^ and interferon-gamma response to mitogen (positive control) in QFT-G test (*P* = .0001). *Conclusions*. Our data suggested that QFT-G had high sensitivity and specificity in HIV-infected populations with CD4^+^ greater than 50/*μ*L. However, QFT-G did not perform well in HIV-positive patients with CD4^+^ less than 50/*μ*L.

## 1. Introduction

Human immunodeficiency virus (HIV) infection is one of the greatest risks for developing active tuberculosis (TB) if HIV-infected individuals are or have been infected with *M. tuberculosis* (MTB). The risk for developing TB in HIV coinfected persons increases approximately 20-to 200-fold compared with immunocompetent individuals [1]. Therefore, chemotherapy for HIV-infected persons with latent TB infection (LTBI) is recommended [1]. Although prevalence of HIV infection in Japan is low (<0.1), the number of HIV-infected persons is increasing year by year [2]. 

In the USA, prophylactic treatment for LTBI has been strongly recommended for HIV-infected persons who have an induration of 5 mm or greater in the tuberculin skin test (TST) [3]. Although the TST has been provisionally proposed as a test for TB infection in HIV-infected subjects in Japan [4], the validity of the TST in this population has not been sufficiently evaluated. This is especially the case in Japan where TST performance is compromised by the past vaccination with Bacillus Calmette-Guerin (BCG) [5], which has been widely used in Japan. 

In 2005, a new diagnostic test for MTB infection, QuantiFERON-TB Gold (QFT-G), was approved in Japan. QFT-G measures T cell responses to *Mycobacterium* RD1-specific antigens, which are absent from BCG vaccine strains and most nontuberculous mycobacteria (NTM) and thereby is more specific than TST [6]. However, as QFT-G measures interferon- (IFN- ) gamma production from T cells responding to the* Mycobacterium *RD1-specific antigens, it is likely that the responses in HIV-infected individuals with lower T cell number would decline. In acknowledgement of this, both the US Centers for Disease Control and Prevention (CDC) and the Japanese Society for Tuberculosis state in their guidelines the necessity for further research on the use of QFT-G in immunocompromised populations, including HIV infected [7, 8]. The CDC guidelines for opportunistic infections in HIV infected persons, issued in April 2009, state that IFN-gamma release assays (IGRAs) such as QFT-G can be used for the diagnosis of LTBI in this population [9]. Although several reports indicate that QFT-G had better performance in diagnosing TB infection in HIV-infected individuals than the TST, sensitivity and specificity vary depending on the setting [10, 11]. In Japan, the only published study of QFT-G in HIV-infected reported a moderate sensitivity of 67% (6/9) in AIDS-TB comorbidity patients; however the size of the study was insufficient to address the general applicability of QFT-G in HIV-infected individuals [12]. 

In the present study, we have examined the usefulness of QFT-G to diagnose MTB infection in HIV-infected individuals as well as comparing the test performance with that of the TST.

## 2. Materials and Methods

### 2.1. Subjects

Inpatients and outpatients of two public general hospitals (Tokyo Metropolitan Fuchu Hospital, currently Tama Medical Center and Komagome Hospital) who were infected with HIV were enrolled into the study regardless of antiretroviral therapy (ART) status. HIV-TB comorbidity was defined as HIV-infected patients with active TB disease. The active TB diagnosis was confirmed by culture positivity in 6 cases. Two other cases were clinically diagnosed based on radiological findings compatible with active TB and their response to antituberculosis treatment. Still another case with tuberculous meningitis was diagnosed so by the elevation of adenosine deaminase in cerebrospinal fluid. 

QFT-G tests were performed together with CD4^+^ T cell count. The clinical history and the demographic data were obtained from the medical chart. When possible, the TST was also performed. Healthy subjects who were known to be not infected with HIV and to have no history of active TB nor MTB exposure were also enrolled as controls. 

This study was approved by the ethics committees of the two hospitals, and informed consent was obtained from all subjects.

### 2.2. Tuberculin Skin Test (TST)

For patients who could revisit their hospital 48 hours after placement for test reading, the TST was performed using the defined standard test dose of tuberculin PPD in Japan (Nippon BCG Manufacturing Co. Ltd, Tokyo, Japan), which is equivalent to 2.5 TU of PPD-S [13], injected intradermally into the volar aspect of the forearm. Transverse induration and erythema diameters were measured 48 to 72 hours later and recorded by trained healthcare workers. Individuals performing and reading the TST were blinded to the QFT-G test results. Induration of 5 mm or greater was interpreted as positive, following the cutoff recommended by the CDC for HIV-positive individuals.

### 2.3. QuantiFERON-TB Gold (QFT)

 The QFT-G assay was performed using QFT-G kits (Cellestis Limited, Carnegie, Australia) according to the manufacturer's instructions. All blood samples were stimulated with *Mycobacterium*-specific antigens within 8 hours of collection. For those subjects who were tested also with the TST, blood collection was done prior to or simultaneously with the QFT-G test. QFT-G results were interpreted according to CDC guidelines [8]. IFN-gamma responses to either ESAT-6 and/or CFP-10 that were greater than or equal to 0.35 IU/mL above the value for the respective Nil control were interpreted as positive. If a person's response (corrected for the Nil control) was less than 0.35 IU/mL for both TB-specific antigens and their response to the mitogen-positive control was above 0.5 IU/mL, they were considered test negative. If the Nil-corrected IFN-gamma response for an individual was less than 0.35 IU/mL for the antigens and less than 0.5 IU/mL for the mitogen-positive control, an indeterminate result was recorded. As per the Japanese interpretation criteria of QFT, a further possible result for QFT-G, “doubtful positive”, was recorded if the subject's response to ESAT-6 and/or CFP-10 was between 0.1 and 0.35 IU/mL and the mitogen response greater than 0.5 IU/mL [14].

### 2.4. Data Analysis

The relationship between the QFT-G results or TST results in association with CD4^+^ T cell count in each patient was analyzed. CD4^+^ T cell count was classified in four categories: less than 50/*μ*L, between 50 and 199/*μ*L, between 200 and 499/*μ*L, and 500/*μ*L and more. QFT-G results were entered into Excel 2003 (Microsoft, Redmond, WA) and transferred to SPSS version 11.0J (SPSS, Inc. Chicago, IL) for statistical analysis. Chi-squared test or Fisher's exact test was used to test the comparison of proportions, and Kruskal-Wallis test was used for testing correlation between IFN-*γ* response to the mitogen and the CD4^+^ T cell count level.

## 3. Results

### 3.1. Characteristics of Patients

A total of 107 cases including 103 Japanese and 4 Thai were enrolled during the study period (Table 1). The majority of the patients were males (92.5%), with a mean age of 46 years (range: 23–75), and mean ± standard deviation of CD4^+^ T cell count was 215 ± 217/*μ*L (range: 4–934). Fifty-one patients were treated with ART, of whom only one patient had the CD4^+^ T cell count below 50. Ninety-eight subjects did not have active TB disease while 2 of these had chest X-ray finding compatible with old TB, and one subject had *M. kansasii *disease. There were 9 subjects with active TB, including one newly infected case who had recent contact with an infectious patient. 

CD4^+^ T cell counts were distributed as shown in Table 1 in patients with or without active TB. The mean cell count tended to be lower for those with TB than those without TB. 

There were 29 healthcare workers (male: 13.8%) with a mean age of 42 years (range: 23–67), recruited as control subjects into the study, and the QFT-G assay was performed for all. They were all negative in the QFT-G assay.

### 3.2. TST in HIV-Infected Patients

Because many of the subjects enrolled into the study were outpatients, they could not return after 48 hours to have their TST read. Thus, the TST was placed for only 26 (24%) and the final results obtained for 23 (21.5%). All subjects with a TST result were Japanese and had been vaccinated with BCG. Of them, 6 had active TB. The TST was positive in 7/23 (30%) patients and negative in 16/23 (70%) (Table 2). The TST positive rate was 4/12 (33%) for those with CD4^+^ T cell count <200/*μ*L, compared with 3/11 (27%) for those with CD4^+^ T cell count more than 200/*μ*L (difference nonsignificant, *P* for Fisher's exact test  =  1.00). 

Six of the 9 HIV-infected patients with active TB had a TST result, and 3 (50%) were positive. One of these TST positive patients had CD4^+^ T cell count less than 50/*μ*L. Of the 17 subjects without TB and with a TST result, 4 were positive, equating to a specificity of 76% (=13/17).

### 3.3. Relationship between CD4^+^ T Cell Count, Presence of Active TB, and QFT-G Results

QFT-G results were available for all of the 107 HIV-infected subjects, and of them 6 (6%) were positive, 92 (86%) negative, and 9 (8%) indeterminate (Table 3). Indeterminate results were significantly associated with very low CD4^+^T cell count, with frequency of indeterminate tests being 25% (8/32) in those with CD4^+^ T cell count less than 50/*μ*L, compared with 1% (1/75) in those with CD4^+^ T cell count greater than 50/*μ*L (Fisher's *P* < .0001). 

For the 9 patients with active TB, 5 (56%) were positive by QFT-G and 1 (11%) indeterminate. There were 3 TB patients with CD4^+^ T cell count less than 50/*μ*L, and QFT-G was negative for two and indeterminate for the other. In contrast, all 5 HIV-TB patients with a CD4^+^ T cell count between 50/*μ*L and 199/*μ*L were QFT-G positive, and the one patient with a CD4^+^ T cell count between 200/*μ*L and 499/*μ*L was negative but the response value was near the cutoff. Of the 98 HIV positive subjects without active TB, one was positive by QFT-G. 

If limiting analysis to those HIV patients with a CD4^+^ T cell count more than 50/*μ*L, the sensitivity of QFT-G for TB infection as seen in TB patients as surrogates of the infected was 83% (5/6), and specificity was 99% (68/69). QFT-G was negative in the two subjects with chest X-ray evidence compatible with old TB and positive in the patient with *M. kansasii *infection. 

 As for ART status, QFT-G was positive in 3 of 5 active TB patients with ART and 2 of 4 cases without ART. In one patient who developed TB within one month after starting ART, QFT-G was positive.

### 3.4. Relationship between CD4^+^ T Cell Count and Positive Control Level in QFT-G

**Figure 1:**
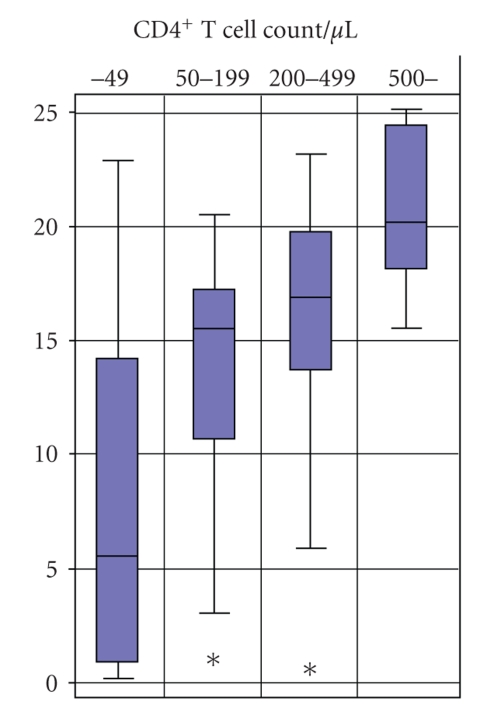
Box plot of the interferon-gamma response to mitogen according to the CD4^+^ T cell count level. ∗ indicates an outlier.

As the QFT-G indeterminate rate was high for HIV patients with CD4^+^ T cell count less than 50/*μ*L as seen above, we analyzed the relationship between CD4^+^ T cell count and level of responses to the test's positive control (stimulation with mitogen) for a total of 95 patients excluding those with TB (*n* = 9) or *M. kansasii* disease (*n* = 1) and those with negative control response being higher than positive control response (*n* = 2). As shown in Figure 1 and Table 4, there is a continuous rise in the response level along with the cell count from less than 50/*μ*L up to over 500/*μ*L with statistical significance (Kruskal-Wallis test, *P* = .0001). There is no significant difference in the level of response between HIV-infected patients with CD4^+^ T cell count greater than 500/*μ*L and healthy control subjects (Table 4).

## 4. Discussion

Although the TST has been used as a diagnostic tool for TB infection for many decades, the specificity of the TST is known to be low in not only HIV-infected individuals but also in the general population of Japan where BCG vaccination is widely carried out. Moreover, TST requires two visits of health care providers for administration and measurement of a test with 48-hour time interval. This is a significant barrier for the cooperation of the patients. In fact, only 21.5% of the enrolled patients in our study underwent a TST in the clinical setting of this study, and the number of cases with TST was not enough for thorough evaluation of TST results. 

The QFT-G was approved in 2005 in Japan, but there remain several issues to be addressed, such as applicability of QFT-G for children or for immunocompromised populations such as HIV infected individuals [15]. Several reports have been published on QFT-G's performance in the HIV infected [10–12], but the present study is the first report which evaluates the QFT-G performance in a large number of HIV-infected individuals in Japan, one of TB middle-burden countries. The data suggest that QFT-G has high sensitivity for TB infection in HIV coinfected patients who have CD4^+^ T cell count > 50/*μ*L, but based on a very small sample size of active TB cases, the test had poor sensitivity in patients with very low CD4^+^ T cell count (<50/*μ*L). 

As would be expected for QFT-G, the test was highly specific in the HIV cohort without active TB, with only one of the 69 non-TB patients being QFT-G positive. The one person who was QFT-G positive had *M. kansasii* infection. This is an expected result as *M. kansasii* is one of the few NTM that carry the RD1 gene which encodes the ESAT-6 and CFP-10 proteins used in QFT-G [16]. In contrast, the TST had a poor specificity of 76% (13/17) in the HIV-positive subjects tested, likely due to the effects of BCG vaccination and revaccination in the Japanese population.

Previous studies of QFT-G indeterminate rates for HIV infected reported that their frequency increased with CD4^+^ T cell count less than 100 or 200/*μ*L [17–20]. Similarly, we found a significant evidence for an increased indeterminate rate in the group of patients with CD4^+^ T cell count less than 50/*μ*L, although the number of each group was not so large. At the same time we found that there is a clear proportional relationship between T cell count and the level of IFN-gamma response in those with cell count less than 500/*μ*L. This implies that HIV-infected patients with T cell count above 50/*μ*L (and less than 500/*μ*L) have also impaired IFN-gamma response more or less although their QFT-G test results are not “indeterminate”. The differences between studies could be due to the relatively small sample sizes, so that cases with slight decrease of response in those with intermediate cell count group could be not judged as “indeterminate” by chance in a small size of observations. Therefore, care should be taken when we interpret the negative QFT-G test results of such subjects, as is the case with the TST. Of course, in such severely immunosuppressed individuals as with CD4^+^ T cell count less than 50/*μ*L, no immunologically based test should be considered as definitive and reliable, and clinicians should use all available information in evaluating MTB infection status. 

Comparison of the performance of QFT-G and TST in diagnosing MTB infection in HIV positive patients was very limited in our study by the small number of patients for whom TST results were available. QFT-G appeared to have at least as good sensitivity as the TST and significantly better specificity, but the number of subjects was insufficient to make definitive conclusions. Of interest was the very low number of subjects for whom TST results were available (23/107). For most people who were not tested by TST, this was due to the requirement to return 48 hours later to have the test read. This highlights a significant benefit of QFT-G—the fact that only one visit to the clinician is required to obtain a result.

There were some limitations in our study. We used the liquid antigen version of the QFT-G test, which has been replaced by the In-Tube version of the test (QFT-GIT) in most countries worldwide. This makes comparisons of our results with those from other studies difficult as most other studies have used QFT-GIT. Since Harada et al. have shown that QFT-GIT has higher sensitivity than QFT-G with the same high specificity [21], it could be expected that the better performance would be obtained than that obtained in this study. The relatively small number of patients with confirmed active TB limited any detailed analysis of sensitivity and the small number of patients for whom TST results were available limited comparison of test performance.

## 5. Conclusion

In HIV-infected individuals, sensitivity and specificity of the TST for the diagnosis of TB infection were poor under the influence of BCG vaccination. In contrast, our data suggested that QFT-G had high sensitivity and specificity in HIV-infected populations with CD4^+^ T cell count greater than 50/*μ*L. However, neither test performed well in HIV-positive patients with CD4^+^ T cell count less than 50/*μ*L. Therefore, care should be taken when interpreting negative or indeterminate QFT-G results in HIV-infected patients with CD4^+^ T cell count less than 50/*μ*L. Further studies in HIV-infected people are required to accumulate more QFT-G performance data in active TB patients in developed countries.

## Figures and Tables

**Table 1:** Characteristics of subjects.

	Cases with active TB	Cases without active TB	Total
Nationality (Japanese/Thai)	8/1	95/3	103/4
Gender (Male/Female)	8/1	91/7	99/8
Mean age (range)	53 (39–65)	45 (23–75)	46 (23–75)
Mean and SD of CD4^+^ T cell count (range)/*μ*L	105 ± 90 (9–270)	226 ± 221 (4–934)	214 ± 215 (4–934)
No. of patients treated with ART	4	44	48 (one interrupted)
TB already treated	0	2	2
Healed TB on chest X-ray	0	2	2
*M. kansasii* disease	0	1	1
N	9	98	107

**Table 2:** Relationship between TST results and CD4^+^ T cell count.

CD4^+^ T cell count/*μ*L	Presence of active TB disease*	TST positive	TST negative
<200 [<50]	Y (*n* = 6)	3 (50%) [1]*	3 (50%) [2]*
	N (*n* = 6)	1 (17%) [0]*	5 (83%)[3]*
≥200	Y (*n* = 0)	0	0
	N (*n* = 11)	3 (27%)	8 (73%)

Y: present, N: absent.

(%): percentage of Y or N number in each CD4 category.

*[  ] indicates the number of those with CD4^+^ T cells less than 50.

**Table 3:** Relationship between QFT-G results and CD4^+^ T cell count.

Category by CD4^+^ T cell count/*μ*L	Active TB	Results of QFT-G			
		Negative	Doubtful positive	Positive	Indeterminate^+^
<50 (*n* = 32)	Y (*n* = 3)	2 (67%)	0	0	1 (33%)
	N (*n* = 29)	22 (76%)	0	0	7 (24%)
50–199 (*n* = 29)	Y (*n* = 5)	0	0	5 (100%)	0
	N (*n* = 24)	23 (96%)	0	1 (4%) a	0
200–499 (*n* = 35)	Y (*n* = 1)	0	1 (10%)	0	0
	N (*n* = 34)	32 (94%)	1 (3%)	0	1 (3%)
≥500 (*n* = 11)	N (*n* = 11)	11(100%)	0	0	0
Total	Y (*n* = 9)	2 (22%)	1 (11%)	5 (56%)	1 (11%)
	N (*n* = 98)	88 (90%)	1 (1%)	1 (1%)	8 (8%)

Y: present, N: absent, and a: *M. kansasii* disease.

(%): percentage of Y or N number in each CD4 category.

^+^Indeterminate results were significantly associated with CD4^+^T cell count less than 50/*μ*L, compared with CD4^+^ T cell count greater than 50/*μ*L (Fisher's *P* < .0001).

**Table 4:** Interferon-gamma responses for mitogen according to CD4^+^ T cell count category in HIV-infected patients without active TB disease and healthy controls.

	<49/*μ*L	50–199/*μ*L	200–499/*μ*L	>500/*μ*L	Healthy controls
Number	27 a	22	35	11	29
Mean	7.48	13.50	16.08	20.40	19.27
S.D.	7.03	5.60	5.17	3.20	5.06
Median	5.55	15.51	16.91	20.21	20.03
Maximum	22.93	20.51	23.19	25.18	26.31
Minimum	0.14	0.67	0.25	15.56	5.12

S.D.: standard deviation.

a. 2 of 29 cases were omitted because negative control value was higher than response to mitogen.
